# Some Notes on the Thermodynamic Accuracy of Coarse-Grained Models

**DOI:** 10.3389/fmolb.2019.00087

**Published:** 2019-09-10

**Authors:** Ewa Anna Oprzeska-Zingrebe, Jens Smiatek

**Affiliations:** Institute for Computational Physics, Theoretical Chemical Physics, University of Stuttgart, Stuttgart, Germany

**Keywords:** coarse-grained (CG) model, thermodynamics, Kirkwood-Buff theory, free energies, implicit solvent model

Over the last decades, multiscale molecular dynamics (MD) simulations including *ab initio*, atomistic as well as coarse-grained models have significantly expanded our understanding of biologically relevant macromolecules like DNA, RNA, or proteins and their properties in solution. Despite the broad applicability, we comment here on some general challenges for coarse-grained approaches, the most important being a reliable thermodynamic description at large time and length scales.

Due to a massive increase in computational power, classical atomistic MD simulations are nowadays the method of choice for the study of complex molecular mechanisms, thereby taking into consideration hundreds of thousands of atoms on time scales of several microseconds. Although classical atomistic models provide a higher level of detail when compared to coarse-grained approaches, it has to be noted that the simplification of electronic behavior in terms of potential functions, so called force fields, introduces some conceptual artifacts into the dynamic and structural properties of the simulated molecular species (Dommert et al., 2012). Furthermore, polarization and charge-transfer mechanisms are usually ignored, such that more sophisticated *ab initio* or empirical models have to be used for systems where these effects become of importance (Smiatek et al., 2018; Kohagen et al., 2019; Nandy and Smiatek, 2019; Smiatek, 2019).

However, some processes take place on time and length scales, which are not accessible for atomistic MD simulations. Common examples are the formation of lipid bilayers and polyelectrolyte complexes, polymer and colloidal diffusion, charge transport or large scale DNA translocation (Smiatek and Schmid, 2011; Michalowsky et al., 2017, 2018; Smiatek and Holm, 2018). For the study of these and closely related problems, simple as well as more refined coarse-grained models offer a wide range of applications. Here, coarse-graining means the introduction of effective interaction sites (beads) instead of individual atoms, which reduces the degrees of freedom and thus also the number of necessary computations. In addition, the lower level of detail supports the straightforward use of implicit solvent approaches in combination with larger time steps (Marrink and Tieleman, 2013; Kleinjung and Fraternali, 2014; Onufriev and Case, 2019). Depending on the degree of coarse graining, one can differentiate between simple approaches such as reduced bead-spring models for polymers and advanced or semi coarse-grained methodologies such as iterative Boltzmann inversion or the MARTINI method among others (Reith et al., 2003; Clark et al., 2012; Marrink and Tieleman, 2013; Noid, 2013; McCarty et al., 2014; Rudzinski and Noid, 2014; Dunn and Noid, 2015; Guenza et al., 2018; Smiatek and Holm, 2018). Although advanced coarse-graining approaches are often based on rather mild parameterization procedures, it should be noted that the consideration of effective interaction sites crucially affects the resulting size and the geometry of the molecular species (Vögele et al., 2015a; Michalowsky et al., 2017, 2018). With regard to this point, also coarse-grained methodologies reveal some generic drawbacks, thereby limiting the applicability of these approaches for the thermodynamic analysis of complex solutions.

In terms of a specific example, many biologically relevant solutions, such as in mammalian or bacterial cells, are dense mixtures of various ions, co-solute and co-solvent species including a non-negligible concentration of solute components (Zhou et al., 2008). Among other effects, the individual components of the solution and their thermodynamic properties exert a tremendous influence on the structural stability of the dissolved biological species (Canchi and García, 2013; Smiatek, 2017; Oprzeska-Zingrebe and Smiatek, 2018a). For instance, it was shown (Zhang and Cremer, 2010; Canchi and García, 2013; Sukenik et al., 2013; Oprzeska-Zingrebe et al., 2018) that ions like SCN^−^ or molecules like urea destabilize DNA or protein structures, whereas the presence of ${\text{SO}}_{4}^{2-}$, trimethylamine-N-oxide (TMAO), or ectoine enhances the stability of native macromolecular states. Additionally, many molecular mechanisms are also dominated by intra- and intermolecular hydrogen bonds, polarization mechanisms as well as electrostatic and dispersion interactions. The presence of these mainly short-ranged interactions influences the radial distribution functions, potentials of mean force or the corresponding chemical potentials of the species, so that in the end, for non-negligible concentrations, there are more or less pronounced deviations from ideal solutions (Chandler, 1987; Smiatek, 2014, 2017; Dunn and Noid, 2015; Guenza et al., 2018; Oprzeska-Zingrebe and Smiatek, 2018a). The question now is whether coarse-grained models can reproduce these findings? Of course, one may wonder if the aforementioned properties need to be exactly reproduced, but we will illustrate by means of the following arguments that even slight deviations may have a decisive influence on the thermodynamic properties of the solution.

In more detail, modified interactions like in coarse-grained models under constant pressure *p* and temperature *T* result in variations of free energies, as defined by *G* = *H* − *TS* with the enthalpy *H* and the entropy *S*, and changes in the chemical potential via μ_α_ = (∂*G*/∂*N*_α_)_*p, T*_ where *N*_α_ denotes the number of molecules of species α. Due to changes in the enthalpy, also the corresponding molecular arrangements are affected, which often induces entropic variations as a second-order effect. Furthermore, changes of chemical potentials from reference chemical potential $\mu_{\alpha }^{0}$ with the universal gas constant *R* are directly related to changes in thermodynamic activities $a_{\alpha }=\text{exp}\left(\left(\mu_{\alpha }-\mu_{\alpha }^{0}\right)/RT\right)$, vapor pressures, solubilities or chemical reaction equilibria, as can be shown by relations from equilibrium thermodynamics and Kirkwood-Buff (KB) theory (Kirkwood and Buff, 1951; Ben-Naim, 2013) . In consequence, it becomes obvious that even slight modifications of molecular interactions may establish a non-negligible variation of relevant thermodynamic properties as it will be discussed in more detail in the following.

For illustrative purposes, we develop our arguments for a binary solution under isobaric-isothermal conditions with two components, including only solvent (index 1) and co-solvent (index 3) species. It has to be noted that the corresponding expressions change for different ensembles and higher-component mixtures, such that we here focus on one of the simplest examples (Smith, 2006). In KB theory, the derivative of the chemical potential of the co-solvent μ_3_ is defined as

(1) $$\begin{array}{r} \frac{1}{RT}{\left(\frac{\partial \mu_{3}}{\partial \text{ ln }\rho_{3}}\right)}_{\text{T},\text{p}}={\left(\frac{\partial \text{ ln }a_{3}}{\partial \text{ ln }\rho_{3}}\right)}_{\text{T},\text{p}}=\frac{1}{1+\rho_{3}\left(G_{33}-G_{31}\right)}, \end{array}$$

where ρ_3_ denotes the number density of co-solvent species and *G*_33_ and *G*_31_ the corresponding KB integrals. A detailed explanation of KB integrals, their relation to radial distribution functions and their central meaning in KB theory can be found in the literature (Kirkwood and Buff, 1951; Ben-Naim, 2013; Smiatek, 2017; Oprzeska-Zingrebe and Smiatek, 2018a). For our considerations, it is sufficient to know that the KB integrals rely on radial distribution functions and represent excess volumes, which can be transformed into excess particle numbers $N_{\alpha \beta }^{\text{xs}}=\rho_{\beta }G_{\alpha \beta }$ for arbitrarily chosen components β around species α. With regard to this definition, Equation (1) can also be written as

(2) $$\begin{array}{r} {\left(\frac{\partial \text{ ln }a_{3}}{\partial \text{ ln }\rho_{3}}\right)}_{\text{T},\text{p}}=\frac{1}{1+\left(N_{33}^{\text{xs}}-\left(\rho_{3}/\rho_{1}\right)N_{31}^{\text{xs}}\right)} \end{array}$$

with the excess number of solvent $N_{31}^{\text{xs}}$ and co-solvent molecules $N_{33}^{\text{xs}}$ in combination with the corresponding number densities ρ_1_ and ρ_3_. In terms of implicit solvent approaches with a continuum dielectric background, it follows that $N_{31}^{\text{xs}}=0$ by definition, which implies that Equation (2) approaches the outcomes of experiments and atomistic models only under nearly ideal conditions with ρ_3_ → 0 at infinite dilution. Further deviations can be observed for large and spherical coarse-grained solvent beads such that the resulting excess volumes are often not correctly reproduced (Vögele et al., 2015a), which implies a significant influence on bulk thermodynamic properties like solubilities or isothermal compressibilities (Pierce et al., 2008; Smiatek et al., 2018).

Noteworthy, also the transfer free energies in ternary mixtures between the co-solvent “3” and the solute “2” as defined by $G^{†}=N_{23}^{\text{xs}}-\left(\rho_{3}/\rho_{1}\right)N_{21}^{\text{xs}}$ rely on accurate values for the number densities and the excess numbers of molecules (Smiatek, 2017; Oprzeska-Zingrebe and Smiatek, 2018b) Otherwise, the thermodynamic affinity between the considered species is crucially affected. In order to highlight some further inconsistencies, it can be shown that also the chemical equilibrium between distinct chemical states in coarse-grained models differs from experimental values and atomistic approaches. In contrast to the chemical equilibrium constant *K*_0_ in presence of a neat solute-solvent mixture, the modified chemical equilibrium constant K^*^ for denatured or native protein or DNA states (Oprzeska-Zingrebe and Smiatek, 2018a,b) or for associated and dissociated ion pairs (Krishnamoorthy et al., 2018) in presence of low co-solvent concentrations reads (Oprzeska-Zingrebe et al., 2019)

(3) $$\begin{array}{r} K^{*}=K_{0}\text{ exp}\left(\Delta N_{23}^{\text{xs}}\right) \end{array}$$

with $\Delta N_{23}^{\text{xs}}=N_{23}^{\text{xs}}\left(d\right)-N_{23}^{\text{xs}}\left(n\right)$ where *d* denotes the denatured and *n* the native state (Oprzeska-Zingrebe et al., 2019). With regard to the previous equation, a different value of $\Delta N_{23}^{\text{xs}}$ as obtained from the coarse-grained simulations when compared to the atomistic model or experimental values ($\Delta N_{23,\text{exp}}^{\text{xs}}$) modifies the chemical equilibrium constant $K^{*}\neq K_{\text{exp}}^{*}$ and also the free energy difference in accordance with $\Delta G^{*}=-RT\ln\;K^{*}\neq \Delta G_{\text{exp}}^{*}$.

In consequence, incorrect sizes and geometries as well as simplified interactions or inaccurately parameterized coarse-grained interaction sites may induce significant deviations and spurious artifacts. A recent article revealed that specifically the number of interaction sites is of crucial importance (Dunn and Noid, 2015). Noteworthy, most deviations are only relevant for small molecular species like organic solvent molecules or ions, whereas significant improvements of coarse-grained models for polymers were recently reported (McCarty et al., 2014; Dunn and Noid, 2015; Vögele et al., 2015a,b; Guenza et al., 2018; Michalowsky et al., 2018).

In terms of these challenges, why should one use coarse-grained models at all? To answer this question, one should keep in mind that everything should be made as simple as possible, but not simpler. As already discussed, deviations between atomistic and coarse-grained models are mainly relevant for small molecular or ionic species where coarse-graining means a significant change of size and geometry. With regard to this point, it was recently shown that improvements in the parameterization strategy, the functional form of the interaction potentials as well as the consideration of polarizabilities in coarse-grained models increase the validity of the results (Noid, 2013; Rudzinski and Noid, 2014; Dunn and Noid, 2015; Michalowsky et al., 2017, 2018; Zeman et al., 2017; Guenza et al., 2018; Uhlig et al., 2018). With regard to this point, variations in thermodynamic properties become even visible for united- and all-atom models which highlights the importance of accurately parameterized molecular structures and interaction sites (Markthaler et al., 2017). Nevertheless, if the key features of interest can be reproduced through reduced models, nothing stands in the way of using these approaches. Otherwise, one must always be aware that uncontrollable artifacts may occur. In consequence, one may always keep the limits of the individual models in mind, such that the applicability of the approaches for certain research questions should be carefully reviewed.

## Author Contributions

EO-Z and JS wrote, reviewed, and edited all versions of this article.

### Conflict of Interest Statement

The authors declare that the research was conducted in the absence of any commercial or financial relationships that could be construed as a potential conflict of interest.
